# Prophylactic Antibiotic Regimens In Tumor Surgery (PARITY): a multi-center randomized controlled study comparing alternative antibiotic regimens in patients undergoing tumor resections with endoprosthetic replacements—a statistical analysis plan

**DOI:** 10.1186/s13063-021-05147-2

**Published:** 2021-03-22

**Authors:** Patricia Schneider, Diane Heels-Ansdell, Lehana Thabane, Michelle Ghert, on behalf of the PARITY Investigators, Michelle Ghert, Mohit Bhandari, Benjamin Deheshi, Gordon Guyatt, Ginger Holt, Timothy O’Shea, R. Lor Randall, Lehana Thabane, Roberto Vélez, Jay Wunder, Patricia Schneider, Victoria Giglio, Paula McKay, Sheila Sprague, Diane Heels-Ansdell, Lisa Buckingham, Peter Rose, Brian Brigman, Eleanor Pullenayegum, Robert Turcotte, David Wilson, Peter Ferguson, Krista Goulding, Joel Werier, Hesham Abdelbary, Paul Clarkson, Marc Isler, Sophie Mottard, Norbert Dion, Annie Arteau, Jennifer Halpern, Herbert Schwartz, Megan Anderson, Mark Gebhardt, Kevin Jones, John Healey, Marcos Galli Serra, Mark Clayer, Adam Lindsay, Tessa Balach, John Abraham, Scot Brown, Benjamin Miller, Edward Cheng, Thomas Scharschmidt, Joel Mayerson, Nickolas Reimer, David Geller, Bang Hoang, Raffi Avedian, Shannon Puloski, Michael Monument, Albert Aboulafia, Timothy Damron, Howard Goodman, Kurt Weiss, Mark Goodman, Joseph Schwab, Matthew DiCaprio, Bradford Palmer, Yee-Cheen Duong, Kenneth Gundle, James Hayden, Carol Morris, Adam Levin, Reitze Rodseth, Leonard Marais, André Mathias Baptista, Juan Pablo Zummaraga, Rosanna Wustrack, Richard O’Donnell, Joel Sorger, Daniel Lerman, André Spiguel, C. Parker Gibbs, Mark Scarborough, Sander Dijkstra, Michiel van de Sande, Shah Alam Khan, Venkatesan Sampath Kumar, John Neilson, Eric Henderson, David Greenberg, Paul Jutte, Nathan Mesko, Lukas Nystrom, Ahmed Elghoneimy, Ricardo Becker, Richard Nicholas, Nicholas Bernthal, Jeffrey Eckhardt, Francis Hornicek, Andreas Leithner, Marko Bergovec, Mann Hong Tan, Suraya Zainul Abidin, Steven Thorpe

**Affiliations:** 1 Department of Surgery, McMaster University, Hamilton, ON L8V 1C3 Canada; 2 Department of Health Research Methods, Evidence and Impact, McMaster University, Hamilton, L8S 4L8 ON Canada; 3 Juravinski Hospital and Cancer Centre, Hamilton Health Sciences, 711 Concession Street, B3 169A, Hamilton, L8V 1C3 ON Canada

**Keywords:** Orthopedic oncology, Bone sarcoma, Randomized controlled trial, Antibiotics, Statistical analysis plan

## Abstract

**Background:**

Limb salvage with endoprosthetic reconstruction is the current standard practice for the surgical management of lower extremity bone tumors in skeletally mature patients and typically includes tumor resection followed by the functional limb reconstruction with modular metallic and polyethylene endoprosthetic implants. However, owing to the complexity and length of these procedures, as well as the immunocompromised nature of patients treated with chemotherapy, the risk of surgical site infection (SSI) is high. The primary research objective of the Prophylactic Antibiotic Regimens In Tumor Surgery (PARITY) trial is to assess whether a 5-day regimen of post-operative antibiotics decreases the risk of SSI at 1 year post-operatively compared to a 1-day regimen. This article describes the statistical analysis plan for the PARITY trial.

**Methods/design:**

The PARITY trial is an ongoing multi-center, blinded parallel two-arm randomized controlled trial (RCT) of 600 participants who have been diagnosed with a primary bone tumor, a soft tissue sarcoma that has invaded the bone or oligometastatic bone disease of the femur or tibia that requires surgical resection and endoprosthetic reconstruction. This article describes the overall analysis principles, including how participants will be included in each analysis, the presentation of results, adjustments for covariates, the primary and secondary outcomes, and their respective analyses. Additionally, we will present the planned sensitivity and sub-group analyses.

**Discussion:**

Our prior work has demonstrated (1) high rates of SSI after the treatment of lower extremity tumors by surgical excision and endoprosthetic reconstruction, (2) highly varied opinion and practice among orthopedic oncologists with respect to prophylactic antibiotic regimens, (3) an absence of applicable RCT evidence, (4) extensive support from international investigators to participate in a RCT, and (5) the feasibility of conducting a definitive RCT to evaluate a 5-day regimen of post-operative antibiotics in comparison with a 1-day regimen.

**Trial registration:**

ClinicalTrials.gov NCT01479283. Registered on 24 November 2011

## Background

Limb salvage surgery is the current standard of care in the management of sarcoma of the long bones [1–3]. Advances in chemotherapeutic regimens and imaging techniques allow for wide resection and functional reconstruction in 95% of patients. The most common type of long-bone reconstruction involves the use of a tumor prosthesis or endoprosthesis. Due to the complexity and length of surgical resection and reconstruction, as well as the immunocompromised nature of patients treated with chemotherapy, the risk of surgical site infection (SSI) remains high, which is a devastating complication that often requires staged revision surgery and long-term intravenous antibiotics [4, 5]. The risk for subsequent infection remains high, as does the risk for ultimate amputation [4, 5]. Moreover, patients’ quality-of-life and function following infection are dramatically impacted, as are healthcare costs [6, 7]. However, the most effective antibiotic regimen in preventing post-operative SSI remains controversial, and the current state of practice varies widely, particularly with respect to antibiotic duration [8]. Strategies to prevent SSIs and optimize quality-of-life while mitigating healthcare costs are needed.

**Fig. 1:**
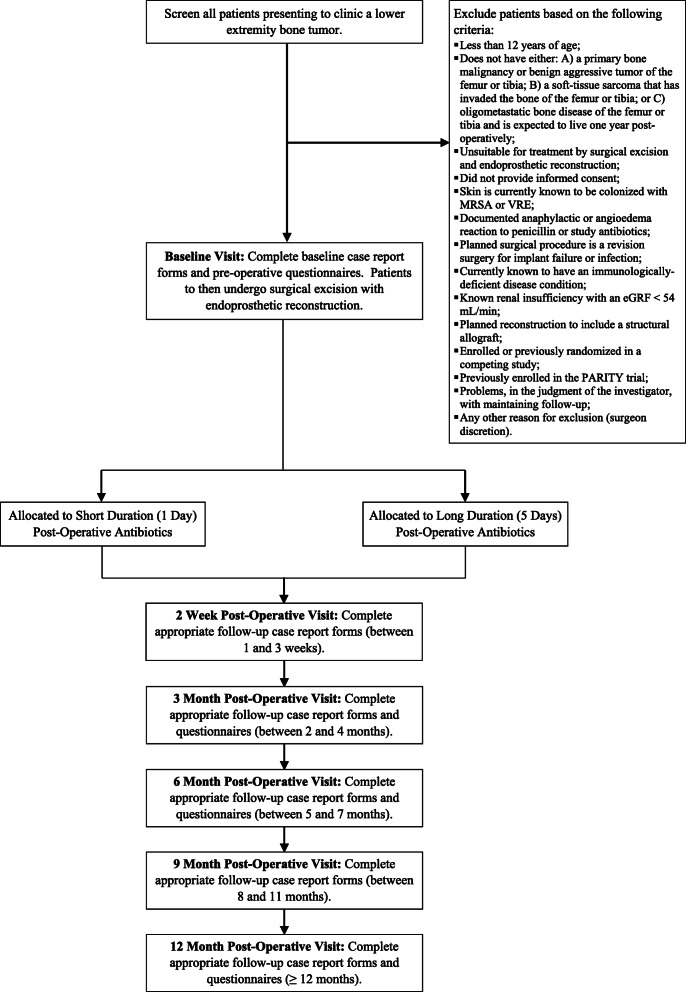
Study process overview

The Prophylactic Antibiotic Regimens In Tumor Surgery (PARITY) trial is an ongoing international, multi-center randomized controlled trial (RCT) using a parallel two-arm design to determine whether a long duration (5 days) of post-operative prophylactic antibiotics decreases the risk of SSI when compared to a short duration (1 day) [9]. The protocol for the PARITY trial has been published elsewhere and provides more detail on the trial rationale, eligibility criteria, interventions, data management, and methods for minimizing bias [9]. Briefly, 600 participants 12 years of age or older undergoing surgical excision and endoprosthetic reconstruction of a lower extremity bone tumor across North America, South America, Europe, Australia, Africa, and Asia are randomized to receive either a short (1 day) or long (5 days) duration of post-operative antibiotics. Allocation is concealed using a centralized and automated 24-h computerized randomization platform that allows Internet-based randomization. Randomization is stratified by tumor location (femur or tibia) and clinical site in randomly permuted blocks of 2 and 4. The primary outcome of the study is the development of a SSI, guided by the Centers for Disease Control and Prevention (CDC) National Healthcare Safety Network reporting criteria [10]. Secondary outcomes include the development of antibiotic-related complications (such as gastrointestinal infections, fungal infections), unplanned re-operations, oncologic outcomes, mortality, and patient functional outcomes and quality-of-life at 1 year. Participants are regularly monitored post-operatively by the treating surgeon at 2 weeks, 6 weeks, 3 months, 6 months, 9 months, and 1 year following surgery. Outcome assessors and data analysts are blinded to treatment allocation. The full study process is shown in Fig. 1.

In this article, we present our planned statistical analyses for the PARITY trial. The statistical analysis plan was finalized and approved on 11 January 2021 (version 1) for the PARITY trial protocol (31 October 2016, version 6) and in accordance with the trial master file, including the data management plan (11 December 2020, version 2). Ethics approval was granted for the Methods Centre at McMaster University (Hamilton Integrated Research Ethics Board No. 12-009) and at each participating clinical site (as per their local ethics committee). This trial is registered on ClinicalTrials.Gov (NCT01479283).

## Methods

### Outcomes

#### Primary outcome

The primary outcome of the PARITY trial is the development of a SSI within 1 year following the initial surgery to treat a lower extremity bone tumor. The primary analysis is to assess whether a long duration regimen (5 days) of post-operative antibiotics decreases the risk of SSI at 1 year compared to a short duration regimen (1 day). SSIs are classified according to the criteria established by the CDC, which defines a SSI as an infection occurring within 30 days following the operative procedure or within 1 year if an implant is in place and the infection appears to be related to the procedure [10]. The SSI can involve any part of the body that is opened or manipulated during the operative procedure but excludes the skin incision, fascia, or muscle layers. The participant must also present with at least one of the following:

- ▪ Purulent drainage from the superficial/deep/organ space incision;
- ▪ Organisms isolated from an aseptically obtained culture of fluid or tissue from the superficial/deep/organ space incision;
- ▪ Superficial/deep/organ space incision that is deliberately opened by a surgeon, attending physician, or other designee and is culture-positive OR not cultured and the participant has at least one of the following signs or symptoms: pain or tenderness, localized swelling, redness, or heat; or
- ▪ Diagnosis of a superficial/deep/organ space incisional SSI by a surgeon or attending physician.

#### Secondary outcomes

Secondary outcomes include the following:

1. Antibiotic-related complications including, but not limited to, *Clostridioides difficile*-associated colitis, opportunistic fungal infections, and indwelling catheter-related sepsis;
2. Unplanned re-operations including, but not limited to, amputation, irrigation and debridement, implant revision, and implant exchange;
3. Oncologic events;
4. All-cause mortality;
5. Physician-derived functional outcome as measured by the Musculoskeletal Tumor Society (MSTS)-87 and MSTS-93 scores; and
6. Self-reported functional outcome as measured by the Toronto Extremity Salvage Score (TESS) survey.

The MSTS-87 score is a standardized scoring system that is completed by an individual on the treatment team (preferably the treating surgeon) and measures physical function after treatment for a musculoskeletal tumor across seven domains: motion, pain, stability, deformity, muscular strength, functional activity, and emotional acceptance. The MSTS-93 score is a standardized scoring system that is also completed by an individual on the treatment team and measures functional outcome after treatment for a musculoskeletal tumor across six domains: pain, function, emotional acceptance, support, walking ability, and gait. The TESS survey is a self-administered evaluation tool that was developed to assess physical function and quality-of-life in patients that have undergone limb salvage surgery for tumors of the extremities. The lower extremity portion of the survey contains 30 questions that are framed to ask about the difficulty experienced by the patient in performing each activity over the previous week. The MSTS-87, MSTS-93, and TESS surveys are all commonly accepted functional scoring systems in the orthopedic oncology literature [11–13].

SSIs, antibiotic-related complications, re-operations, and mortality will be reviewed by an independent Adjudication Committee.

### Sample size

At the onset of the trial, we calculated that the definitive trial’s sample size would include 460 participants per group, for a total of 920 participants. This sample size was based on a between-group comparison for the primary outcome of deep SSI following long duration (5 days) or short duration (1 day) prophylactic antibiotics and was calculated to ensure that the study would have a power of 80% to identify differences among the two groups at an alpha level of 0.05, on the basis of an overall event rate of 10% and a presumed 50% or greater reduction in the risk of deep SSI within 1 year.

Prior to the transition from the pilot phase to the definitive phase of the trial, we met with our Steering Committee to finalize the definitive study protocol and processes. At this time, we decided to expand the trial’s primary outcome from deep SSI to any SSI (superficial/deep/organ space SSI) in order to increase the expected event rate and study power without compromising clinical importance. This adjustment in the primary outcome resulted in an overall pilot phase event rate of 14%, which exceeds the overall event rate of 10% used to calculate the initial sample size. As a result, the definitive trial’s sample size was reduced to 300 participants per arm, for a total of 600 participants to identify the differences among the two groups at an alpha level of 0.05 and to ensure that the study would have a power of 80% using the updated event rate of 14% while maintaining the presumed 50% or greater reduction in the risk of SSI within 1 year.

The current sample size calculation is the standard method to determine sample size in a binary outcome study and will provide a conservative yet similar estimate to the more complicated and complex calculation for a time-to-event analysis. This decision was made as utilizing the more conservative binary outcome estimate would likely account for any unforeseen losses to follow-up, dropouts, and crossovers, which were considered negligible in our study population, and therefore, adjustments for their occurrences were not warranted at the time of the definitive sample size calculation. The binary outcome study method is also simpler to present in study documents such as grant applications.

## Discussion

### Analysis plan

This statistical analysis plan follows the JAMA Guidelines for the Content of Statistical Analysis Plans in Clinical Trials [14]. A summary of all planned analyses is provided in Table 1.

**Table 1:** Summary of the statistical analysis plan

Objective	Outcome		Hypothesis	Method of analysis
	Name	Variable type		
Primary objective				
To compare the surgical site infection rates at 1 year	Surgical site infection	Binary	A longer duration (5 days) of post-operative antibiotics will reduce the risk of surgical site infection compared to a shorter duration (1 day) of post-operative antibiotics.	Cox proportional hazards
Secondary objectives				
To compare the risk of antibiotic-related complications at 1 year	Antibiotic-related complications	Binary	A longer duration (5 days) of post-operative antibiotics will result in more antibiotic-related complications compared to a shorter duration (1 day) of post-operative antibiotics.	Cox proportional hazards
To compare the risk of unplanned re-operations at 1 year	Unplanned re-operations	Binary	A longer duration (5 days) of post-operative antibiotics will result in fewer unplanned re-operations compared to a shorter duration (1 day) of post-operative antibiotics.	Cox proportional hazards
To compare the risk of oncologic events at 1 year	Oncologic events	Binary	There will be no difference in the number of oncologic events irrespective of post-operative antibiotic duration.	Cox proportional hazards
To compare the mortality at 1 year	Mortality	Binary	There will be no difference in the risk of death irrespective of post-operative antibiotic duration.	Cox proportional hazards
To compare the patient functional outcomes at 1 year	MSTS-87, MSTS-93	Continuous	A longer duration (5 days) of post-operative antibiotics will result in better patient functional outcomes compared to a shorter duration (1 day) of post-operative antibiotics.	Multiple linear regression
To compare the patient quality-of-life outcomes at 1 year	TESS	Continuous	A longer duration (5 days) of post-operative antibiotics will result in better patient quality-of-life outcomes compared to a shorter duration (1 day) of post-operative antibiotics.	Multiple linear regression
Sub-group analyses				
Tumor type (bone sarcoma, soft tissue sarcoma, and oligometastatic bone disease)	Surgical site infection	Binary	There will be no difference between the tumor types in the association between surgical site infection and post-operative antibiotic duration.	Cox proportional hazards
Tumor location (femur or tibia)	Surgical site infection	Binary	A longer duration (5 days) of post-operative antibiotics will be more effective at reducing surgical site infections relative to a shorter duration (1 day) of post-operative antibiotics in tibial reconstructions than in femoral reconstructions.	Cox proportional hazards
Sex (male or female)	Surgical site infection	Binary	There will be no difference between the sexes in the association between surgical site infection and post-operative antibiotic duration.	Cox proportional hazards
Age (pediatric and young adults [12–30 years of age] or older adults [≥ 31 years of age])	Surgical site infection	Binary	A longer duration (5 days) of post-operative antibiotics will be more effective at reducing surgical site infections relative to a shorter duration (1 day) of post-operative antibiotics in the older adult population than in the pediatric and young adult population.	Cox proportional hazards
Peri-operative chemotherapy	Surgical site infection	Binary	A longer duration (5 days) of post-operative antibiotics will be more effective at reducing surgical site infections relative to a shorter duration (1 day) of post-operative antibiotics in patients who received chemotherapy than in those who did not receive chemotherapy.	Cox proportional hazards
Sensitivity analyses				
Competing risks (death and amputation)	Surgical site infection	Binary	We do not expect the association between post-operative antibiotic duration and surgical site infections to change substantially once we take into account the competing risk of death.	Competing risks analysis
Trial site (center-effects)	Surgical site infection	Binary	We do not expect the results to change substantially when center-effects are removed from the primary analysis.	Cox proportional hazards (with center-effects removed)
Potential prognostic imbalances at baseline	Surgical site infection	Binary	Results will remain robust after adjusting for the following prognostic baseline imbalances: total operative time, tumor location, diabetes status, chemotherapy regimen, and radiation treatment.	Cox proportional hazards

#### Overview

All outcome analyses will be performed using the intention-to-treat (ITT) principle. As a result of stratification, all analyses will be adjusted for tumor location (femur or tibia) and clinical site. The primary analysis will compare the treatment groups on the SSI outcome, and the secondary analysis will compare the treatment groups on the following outcomes at follow-up: antibiotic-related complications, unplanned re-operations, oncologic outcomes, mortality, and patient functional outcomes and quality-of-life. For all models, the results will be expressed as hazards ratios (HRs) for time-to-event outcomes and mean difference for continuous outcomes, with corresponding two-sided 95% confidence intervals (CIs) and associated *p* values. All statistical tests will be performed using two-sided tests at the 0.05 level of significance. Analyses of secondary outcomes are exploratory in nature and, therefore, alpha values will not be adjusted for multiple testing. *p* values will be reported to three decimal places with values less than 0.001 reported as < 0.001. All analyses will be performed using SAS 9.4 (Cary, NC, USA).

#### Blinded analyses

All statistical analyses will first be conducted using blinded treatment groups (i.e., antibiotic duration X and duration Y). To do so, the blinded study statistician will provide complete blinded results labeled antibiotic duration X and duration Y; the remainder of the study team at the Methods Centre will be left unaware of which treatment groups antibiotic durations X and Y represent. Interpretations for the effect of the antibiotic durations will be documented during a blinded review of the data based upon blinded antibiotic duration X versus Y (i.e., we will determine how to interpret the results if antibiotic duration X proves to be the long duration regimen (5 days) of post-operative antibiotics versus how to interpret the results if antibiotic duration Y proves to be the long duration regimen (5 days) of post-operative antibiotics) [15]. We will unblind the results by breaking the randomization code following the approval and documentation of the interpretations by the study team. These agreed-upon interpretations will guide the discussion section of the subsequent definitive trial manuscript.

#### Presentation of data

**Fig. 2:**
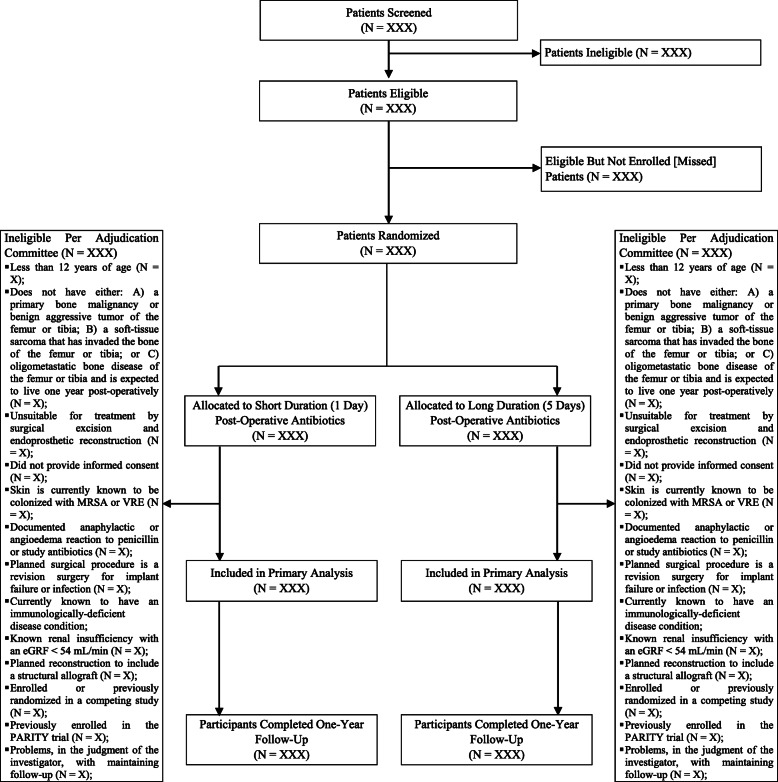
Screening and enrollment flow diagram

The trial results will be presented according to the Consolidated Standards of Reporting Trials (CONSORT) guidelines for RCTs [16]. The number of patients screened, included, and excluded will be presented in a flow diagram (Fig. 2). The baseline demographic characteristics, tumor details, and surgical and peri-operative management characteristics of the participants, as well as details of the prophylactic study antibiotic administrations, will be summarized by group. Continuous data will be presented with means and standard deviations [SD], or medians and first and third quartiles [Q1, Q3] for skewed data, and categorical data will be presented as frequencies and proportions (see Tables 2, 3, 4, and 5).

**Table 2:** Participant demographics and baseline details

Characteristic	Treatment X *N* = XXX	Treatment Y *N* = XXX
Age [years], mean (SD)		
Gender, *n* (%)		
Male		
Female		
Ethnicity, *n* (%)		
White/Caucasian		
Black		
Native		
Asian		
Hispanic		
Others (specify)		
Pre-diagnosis employment, *n* (%)		
Employed		
Not employed		
Retired		
Student		
Homemaker		
Doctor’s advice/disability		
Unemployed		
Other known malignancies at baseline, *n* (%)		
No		
Yes		
Systemic metastases at baseline, *n* (%)		
No		
Yes		
Pulmonary		
Skeletal		
Other viscera (specify)		
Others (specify)		
Other cancer modalities at baseline, *n* (%)		
No		
Yes		
Pre-operative chemotherapy		
Pre-operative radiation		
Others (specify)		
Smoking status, *n* (%)		
Current smoker		
Former smoker		
Never smoked		
Alcohol consumption, *n* (%)		
No		
Yes		
Recreational drug use, *n* (%)		
No		
Yes (specify)		
Diabetes status, *n* (%)		
No		
Yes		
Insulin-dependent (type I)		
Non-insulin-dependent (type II)		
Medication use at baseline, *n* (%)		
None		
NSAIDs		
Analgesics: opioids		
Anti-hypertension medications		
General cardiac medications		
Pulmonary medications		
Osteoporosis medications		
Antibiotics

**Table 3:** Tumor details

Characteristic	Treatment X *N* = XXX	Treatment Y *N* = XXX
Location of tumor, *n* (%)		
Femur		
Tibia		
Location in bone, *n* (%)		
Proximal		
Mid-shaft		
Distal		
Others (specify)		
Maximum size [cm], mean (SD)		
No. of compartments, *n* (%)		
0		
1		
2		
3		
4		
Type of biopsy performed, *n* (%)		
None		
Open		
Fine-needle aspiration		
Core needle		
Type of tumor, *n* (%)		
Bone sarcoma		
Soft tissue sarcoma		
Metastatic bone disease		
Overall margins, *n* (%)		
Negative		
Microscopically positive		
Grossly positive

**Table 4:** Surgical and peri-operative management details

Characteristic	Treatment X *N* = XXX	Treatment Y *N* = XXX
Surgical details		
Length of procedure [min], mean (SD)		
Type of skin sterilization, *n* (%)		
Iodine		
Alcohol		
Others (specify)		
Length of incision [cm], mean (SD)		
Laminar flow, *n* (%)		
No		
Yes		
Spacesuit worn, *n* (%)		
No		
Yes		
Type of resection, *n* (%)		
Intra-articular		
Extra-articular		
Length of bone resected, *n* (%)		
< 5 cm		
5–10 cm		
> 10 cm		
Skin excised, *n* (%)		
None		
Small (< 5 cm^2^)		
Moderate (5–10 cm^2^)		
Large (> 10 cm^2^)		
Muscle excised, *n* (%)		
Small (< 50 cm^3^)		
Moderate (50–100 cm^3^)		
Large (> 100 cm^3^)		
Fascial tissue excised, *n* (%)		
None		
Small (< 1 cm^2^)		
Moderate (1–5 cm^2^)		
Large (> 5 cm^2^)		
Type of fixation, *n* (%)		
Press-fit		
Cement		
With antibiotic		
Without antibiotic		
Cerclage		
Wire		
Cable		
Synthetic		
Bone grafting performed, *n* (%)		
No		
Yes		
Synthetic bone graft		
Autograft		
Cortical		
Cancellous		
Vascularized cancellous		
Allograft		
Cortical		
Cancellous		
Vascular reconstruction, *n* (%)		
No		
Yes		
< 5 cm		
5–10 cm		
> 10 cm		
Intra-operative prophylaxis, *n* (%)		
No		
Yes		
IV heparin		
Tranexamic acid		
Others (specify)		
Antibiotic or silver-coated prosthesis, *n* (%)		
No		
Yes		
Antibiotic (specify)		
Silver		
Antibiotic impregnated sponge or antibiotic powder implanted, *n* (%)		
No		
Yes		
Gentamicin		
Tobramycin		
Cefazolin		
Vancomycin		
Others (specify)		
Irrigation performed at the end of the procedure, *n* (%)		
No		
Yes, pulsed irrigation		
Yes, antibiotics in irrigation		
Mode of skin closure, *n* (%)		
Primary closure		
Local fasciocutaneous flap		
Local muscle flap and split		
Thickness skin graft		
Free flap		
Peri-operative management details		
Post-operative thromboprophylaxis, *n* (%)		
No		
Yes		
Coumadin		
Heparin		
Fractionated heparin		
Oral		
Suction drain, *n* (%)		
No		
Yes		
Duration		
Urinary catheter, *n* (%)		
No		
Yes		
Duration		
No. of patients in a hospital room, *n* (%)		
1		
2		
3		
4		
> 4		
Time to first post-operative wound dressing change [days], mean (SD)		
Negative-pressure wound therapy (wound vac), *n* (%)		
No		
Yes		
Duration		
Length of post-operative hospital stay [days], mean (SD)		
Discharge location, *n* (%)		
Home		
Rehabilitation facility		
Other hospitals		
Others (specify)

**Table 5:** Prophylactic antibiotic administration details

Characteristic	Treatment X *N* = XXX	Treatment Y *N* = XXX
Pre-operative study antibiotic administered per protocol, *n* (%)		
No		
Yes		
Additional pre-operative prophylactic antibiotic(s) administered, *n* (%)		
No		
Yes		
Intra-operative study antibiotic administered per protocol, *n* (%)		
No		
Yes		
Additional intra-operative prophylactic antibiotic(s) administered, *n* (%)		
No		
Yes		
Post-operative study antibiotic/placebo administered per protocol, *n* (%)		
No		
Yes		
Additional post-operative prophylactic antibiotic(s) administered, *n* (%)		
No		
Yes		

#### Primary outcome analysis

Our hypotheses for the primary analysis are as follows:

Null hypothesis: There is no difference in the risk of SSI at 1 year between the two treatment groups.

Alternate hypothesis: There is a difference in the risk of SSI at 1 year between the two treatment groups.

The primary analysis will be a Cox proportional hazards analysis with time from surgery to the SSI as the primary outcome. Post-operative prophylactic antibiotic duration (treatment group [1 day versus 5 days]) will be the independent variable, and the Cox regression will also include tumor location (femur or tibia) and clinical site as stratification variables. All clinical sites with fewer than five participants enrolled will be collapsed into a single clinical site when included in our regression model. Participants who did not experience the primary endpoint will be censored at 12 months or the time of the last visit. The proportional hazards assumption of the Cox model will be assessed by examining Schoenfeld residuals. If the independent variable does not meet the assumption of the proportional hazards, we will modify the model to allow the HR to differ throughout the study period guided by the observed data. Results will be reported as HRs with the corresponding 95% CI and associated *p* values. Kaplan-Meier curves will be constructed for the two randomized treatment groups. For each treatment group, we will also report superficial SSI, deep SSI, and organ space SSI. The results of the primary analysis will be presented in Table 6.

**Table 6:** Study outcomes by treatment group

Study endpoint	Treatment X *N* = XXX *n* (%)	Treatment Y *N* = XXX *n* (%)	Hazard ratio (95% CI)	*p* value
Primary outcome				
Any surgical site infection				
Superficial incisional				
Deep incisional				
Organ/space				
Secondary outcomes				
Any antibiotic-related complication				
Stomach cramps				
Nausea/vomiting				
Oral candidiasis				
Unusual bleeding/bruising				
Difficulty breathing				
Sore mouth/throat				
Allergic reaction (itching, drug fever, skin rash, anaphylaxis)				
Anemia/low blood counts				
Skin reaction				
Diarrhea				
Liver toxicity				
Kidney toxicity				
*Clostridioides difficile*-associated colitis				
Toxic megacolon				
Opportunistic fungal infection				
Indwelling catheter-related sepsis				
Other antibiotic-related events				
Any unplanned re-operation				
Implant revision				
Irrigation and debridement				
Wound flap				
Skin graft				
Bone graft				
Implant exchange				
Extensor mechanism reconstruction				
Repeat tumor excision				
Antibiotic spacer insertion				
Patellar reconstruction				
Abductor reconstruction				
Rotationplasty				
Amputation				
Other unplanned re-operations				
Any oncologic event				
Local recurrence				
Distant metastases				
Mortality due to any cause				
Mortality due to disease progression				

#### Secondary outcomes analysis

We will estimate the effect of post-operative prophylactic antibiotic duration (1 day versus 5 days) on antibiotic-related complications, re-operations, oncologic events, and all-cause mortality at 1 year (Table 6). Similar to the primary analysis, we will perform a Cox proportional hazards analysis. We will only perform Cox regressions for individual antibiotic-related complications, unplanned re-operations, and oncologic and mortality events if there are enough events. Should there be an insufficient number of events, we will summarize by treatment group and report using descriptive statistics (frequencies and proportions). We will also separately report 4-week, 3-month, and 1-year mortality figures for future comparison with other studies.

In addition, we will also estimate the effect of post-operative prophylactic antibiotic duration on patient functional outcomes (MSTS-87 and MSTS-93 surveys) and quality-of-life (TESS surveys) at 1 year (Table 7). To do so, we will use multiple linear regression models that include the following independent variables: randomized treatment group, tumor location (femur versus tibia), clinical site, and baseline score. The results will be reported as the mean differences with 95% CIs. Given that functional and quality-of-life outcomes are the most difficult to collect and, therefore, we expect some missing data, we will use multiple imputation to address the missing data for these outcomes should the amount of missing data be considerable but not too substantial. Convention dictates that if more than five but less than 40% of data is missing, the use of multiple imputation is appropriate and warranted [17].

**Table 7:** Functional and quality-of-life outcomes by treatment group

Study endpoint	Treatment X *N* = XXX Mean (SD)	Treatment Y *N* = XXX Mean (SD)	Mean difference (95% CI)	*p* value
Functional and quality-of-life outcomes				
MSTS-87				
MSTS-93				
TESS				

#### Sensitivity analyses

Sensitivity analyses will be performed for the primary outcome only [18]. We will conduct a competing risks analysis that accounts for deaths and amputation as competing risks. We will also perform sensitivity analyses for center-effects where we will redo the primary analysis without including the clinical site in the model. We will also look for prognostic imbalances between the two treatment groups based on the following key variables known to be risk factors for a SSI: total operative time, tumor location, diabetes status, chemotherapy regimen, and radiation treatment. We will complete adjusted analyses to address any possible baseline imbalance between the groups.

#### Sub-group analyses

**Fig. 3:**
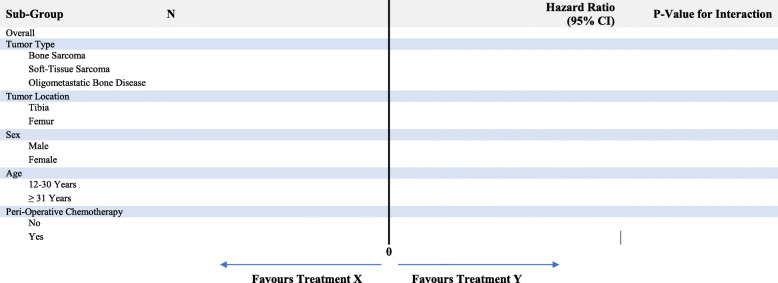
Sub-group analyses of the primary endpoint according to the treatment group

At the onset of the PARITY trial, we identified two important sub-groups (tumor type and tumor location), which will be reported according to the standard guidelines [19]. As we near the end of the trial, prior to unblinding, we have identified a further three important sub-groups (sex, age, and peri-operative chemotherapy). We will add a main effect for the sub-group variable and the treatment by sub-group interaction to our primary model described above to assess whether the magnitude of the treatment effect is significantly different between the sub-groups (Fig. 3). This will be repeated separately for each sub-group variable. We will perform the following sub-group analyses with the primary endpoint as the outcome (Fig. 3):

1. Tumor type—the type of tumor will be classified as follows: bone sarcoma, soft tissue sarcoma, or oligometastatic bone disease. We hypothesize that there will be no difference between the tumor types with regard to the association between prophylactic antibiotic duration and risk of infection.
2. Tumor location—the location of the tumor will be classified as follows: femur or tibia (we will not include the stratification variable of tumor location in this analysis). We hypothesize that a long duration (5 days) of prophylactic antibiotics will be more effective relative to a short duration (1 day) in tibial reconstructions than in femoral reconstructions.
3. Sex—sex will be classified as follows: male or female. We hypothesize that there will be no difference between the sexes with regard to the association between prophylactic antibiotic duration and risk of infection.
4. Age—age will be classified as follows: pediatric and young adults (12–30 years of age) or older adults (≥ 31 years of age). We hypothesize that a long duration (5 days) of prophylactic antibiotics will be more effective relative to a short duration (1 day) in the older adult population than in the pediatric and young adult population.
5. Peri-operative chemotherapy—peri-operative chemotherapy will be classified as follows: no chemotherapy or chemotherapy (neoadjuvant or adjuvant or a combination of the two). We hypothesize that a long duration (5 days) of prophylactic antibiotics will be more effective relative to a short duration (1 day) in patients who received chemotherapy than in those who did not receive chemotherapy.

Rather than pre-specifying a threshold *p* value for making a sub-group claim, we will use the approach suggested by Sun et al. to consider the plausibility of any possible sub-group effects [20]. If a plausible sub-group effect is found, we will further explore the impact of the sub-group on the secondary outcomes. However, due to inadequate sample size and power to conduct the sub-group analyses, these results will be used solely for the generation of hypotheses for further investigations.

#### Interim analyses

No interim analyses are planned due to our desire to avoid spuriously inflated estimates of treatment effects [21, 22]. The PARITY Data and Safety Monitoring Board (DSMB) regularly meets to monitor the study data for participant safety.

#### Dissemination

Upon trial completion, the primary manuscript with the 1-year follow-up results, whether positive, negative, or neutral, will be submitted for peer review and publication in a top-tier medical journal. The final dataset will be shared through an open access data repository once all analyses are completed.

## Trial status

The PARITY trial began as a pilot of 60 participants in January 2013 [23]. Upon demonstrating study feasibility and securing definitive funding (July 2014), these participants were rolled into the definitive study (*N* = 600). Recruitment for the definitive study was completed in October 2019, and the final 1-year follow-up data is expected to be completed and collected in December 2020.

## Data Availability

The final dataset will be shared through an open access data repository once all analyses are completed.

## Abbreviations

CDC: Centers for Disease Control and Prevention
CI: Confidence interval
CONSORT: Consolidated Standards of Reporting Trials
DSMB: Data and Safety Monitoring Board
HR: Hazards ratio
ITT: Intention-to-treat
MSTS: Musculoskeletal Tumor Society
PARITY: Prophylactic Antibiotic Regimens In Tumor Surgery
RCT: Randomized controlled trial
TESS: Toronto Extremity Salvage Score
SD: Standard deviation
SSI: Surgical site infection
